# A Serious Game About Hematology for Health Care Workers (SUPER HEMO): Development and Validation Study

**DOI:** 10.2196/40350

**Published:** 2023-02-13

**Authors:** Julien Perrin, Amélie Meeus, Julien Broseus, Pierre-Jean Morieux, Valentine Di Ceglie, Julien Gravoulet, Maud D'Aveni

**Affiliations:** 1 Faculté de Pharmacie Université de Lorraine Nancy France; 2 Centre Hospitalier Régional Universitaire Nancy France; 3 Faculté de Médecine, Maïeutique et Métiers de la Santé Université de Lorraine Vandoeuvre-Lès-Nancy France

**Keywords:** educational technology, hematology, health care students, education, teaching, validation, methodological study, video support tool, continuing education, serious games, educational games

## Abstract

**Background:**

Complete blood count (CBC) and hemostatic screening tests are among the most commonly prescribed blood tests worldwide. All health care workers (nurse practitioners, pharmacists, dentists, midwives, and physicians) are expected to correctly interpret the results in their daily practice. Currently, the undergraduate hematology curriculum consists predominantly of lecture-based teaching. Because hematology combines basic science (blood cells and hemostasis physiology) and clinical skills, students report that they do not easily master hematology with only lecture-based teaching. Having interviewed students at the University of Lorraine, we considered it necessary to develop new teaching approaches and methods.

**Objective:**

We aimed to develop and validate a serious game about CBC analysis for health care students. Our primary objective was to help students perceive hematology as being a playful and easy topic and for them to feel truly involved in taking care of their patients by analyzing blood tests. We considered that this game-based approach would be attractive to students as an addition to the classic lecture-based approach and improve their knowledge and skills in hematology.

**Methods:**

We developed an adventure game called SUPER HEMO, a video game in which the player assumes the role of a protagonist in an interactive story driven by exploration and problem-solving tests. Following validation with beta testing by a panel of volunteer students, we used a novel, integrated teaching approach. We added 1.5 hours of gaming to the standard curriculum for a small group of volunteer students. Physician and pharmacy students in their third year at a single French university were invited to attend this extracurricular course. Pregame and postgame tests and satisfaction surveys were immediately recorded. Final hematology exam results were analyzed.

**Results:**

A total of 86 of 324 physician students (26.5%) and 67 of 115 pharmacy students (58%) opted to participate. Median scores on the pre- and posttests were 6 out of 10 versus 7 out of 10, respectively, for the physician students, (*P*<.001) and 7.5 out of 10 versus 8 out of 10, respectively, for the pharmacy students (*P*<.001). At the final hematology evaluation, physician students who played SUPER HEMO had a slightly better median score than those who did not: 13 out of 20 versus 12 out of 20, respectively (*P*=.002). Pharmacy students who played SUPER HEMO had a median score of 21.75 out of 30; this was not significantly different from pharmacy students who did not play SUPER HEMO (20/30; *P*=.12). Among the participants who answered the survey (n=143), more than 86% (123/143) believed they had strengthened their knowledge and nearly 80% (114/143) of them had fun.

**Conclusions:**

Feedback from this game session provided evidence to support the integration of interactive teaching methods in undergraduate hematology teaching. The development of SUPER HEMO is intended to be completed so that it can become a support tool for continuing education.

## Introduction

Numerous serious games (SGs) have been developed to improve nursing [1] and medical [2] knowledge and skills. The main subjects are surgery [3-5], emergency medicine [6,7], pharmacy [8] for health care students, and other subjects, such as preventive medicine for adolescents and young people [9-12]. Systematic reviews of SGs conclude that they seem to be at least as effective as other digital education modalities [13], but pedagogical effectiveness, participant behavior, and patient health outcomes have barely been evaluated [14-16].

In the context of the global COVID-19 pandemic, the demand for online learning increased worldwide. However, it has been reported that reduced peer and teacher interaction can cause motivation issues [17,18]. Recently, online learning has been described negatively, especially by health care students in clinical practice [19]. Many health care students see working in the health care setting as a vocation rather than a job, with patient-centered and compassionate care being the basis of this view [20]. Therefore, video games that enable specifically situated, experiential learning by introducing different unwell characters represent an attractive learning tool for today’s students, who are very receptive to computer-based learning.

SGs for hematology education are rare and deal only with transfusion [21,22]. One important part of hematology education is complete blood count (CBC). CBC is a quantitative and qualitative evaluation of blood cells and stands as one of the most common laboratory tests in medicine, being indicated for a vast number of conditions. CBC interpretation is taught only with lecture-based courses in hematology. An internal survey among physician and pharmacy students at the University of Lorraine revealed that more than 50% of students considered CBC interpretation hard to master. Because correctly applying CBC knowledge to form patient diagnoses and make clinical decisions is a requirement for all health professionals, we decided to implement an SG for hematology education.

**Figure 1:**
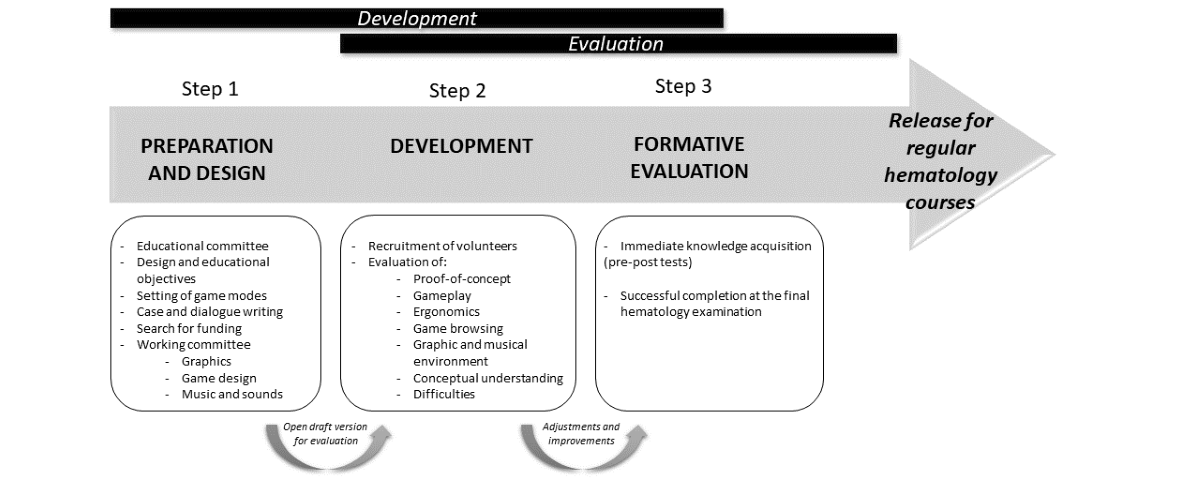
SUPER HEMO development.

We developed an SG (Figure 1) named SUPER HEMO with a structured, 3-phase development framework that included preparation and design, development, and formative evaluation, as described previously [23].

## Methods

### Preparation and Design

#### Framework

As a reference for the creation and development of the game, we took inspiration from role-playing and adventure games (eg, point-and-click games and visual novels), as these are commonly played at home by students. The game’s contents, including a database with questions, answers, and feedback, were based on knowledge from the Collège national des enseignants en hématologie (the French national college of hematology teachers).

At first, we created an educational committee composed of 3 hematology experts (1 pharmacist and 2 medical doctors), a pharmacist with gamification expertise (with no expertise in hematology), and an instructional designer. Meetings took place every month for the first 6 months of the project. The goals of this committee were to define pedagogical objectives, game design, and game modes, and to write the clinical cases’ dialogue. During the writing of the clinical cases, physician students volunteered to test some of the cases before their integration into the game. The committee also looked for funding. The raised funds were mainly used to hire a graphics designer and a game developer.

Then, a working committee was created with 2 medical doctors, a pharmacist with hematology expertise, a pharmacist with gamification expertise, an instructional designer, a graphics designer, and a game developer. Meetings occurred every 2 months to discuss practical issues arising from game conception. Some members worked together outside of the meetings in groups, such as the graphics designer with the hematology expert or the game developer with the instructional designer and the hematology expert.

The project was then presented to the pedagogical council of each faculty.

#### Story

SUPER HEMO is set in a dreamlike “Red Cell World” (Multimedia Appendix 1), with red-blood-cell trees and depictions of the organs involved in erythropoiesis, including a “lung mountain,” “medullar cave,” “kidney rock,” “spleen fortress,” and “thyroid isthmus.”

Players can choose a female or a male avatar on the home screen. They then complete the introduction, in which “Lady Stem Cell” explains the world and the game’s settings and instructions and gives the player the “CBC asset,” which is the power to check the blood parameters of the unwell characters encountered and interpret the corresponding results. Players assume the role of a hematology superhero named SUPER HEMO. SUPER HEMO can meet 5 unwell characters in 5 different steps. The player must answer their questions and find the best way to diagnose and cure them. “Magicians” (radiologists, pathologists, hematology-biologists, pharmacists, and geneticists) can be summoned to help the player find the right answer (Multimedia Appendix 2) in exchange for gold coins.

#### Mechanics

**Figure 2:**
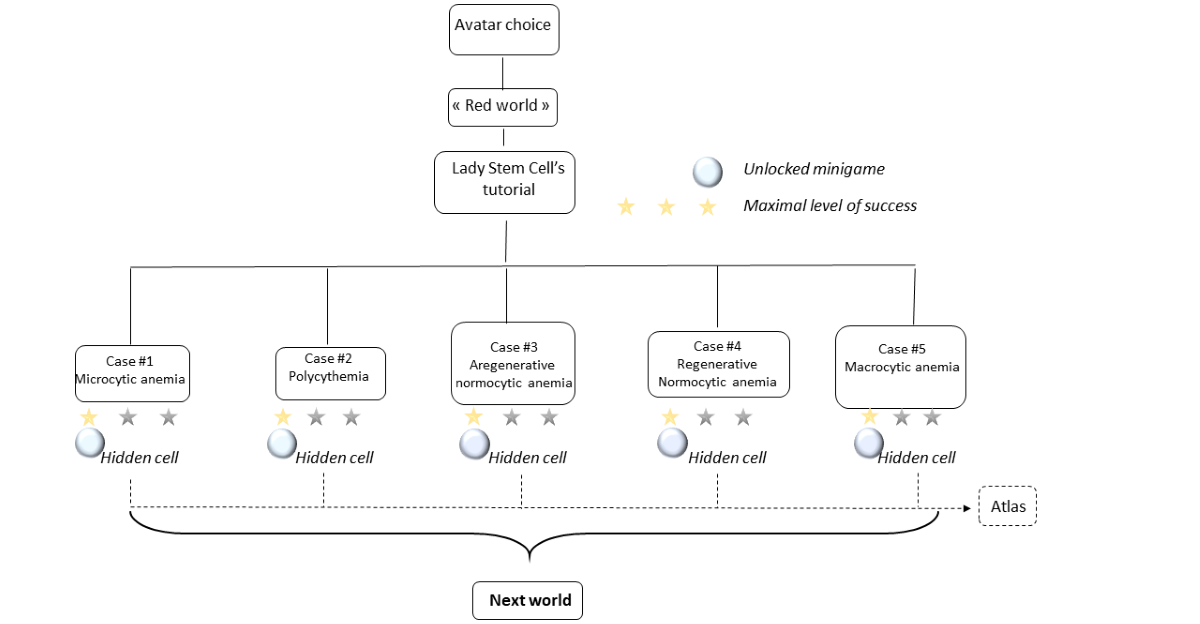
"Red World" mechanics.

To complete the world’s challenges (Figure 2), SUPER HEMO must explore 5 clinical cases.

Each stop (represented by a light blue button) corresponds to the clinical case of an unwell character. Players choose to explore the clinical cases in the order that they decide. When SUPER HEMO successfully ends a case, players are rewarded with 1, 2, or 3 stars depending on the number of errors they made. Moreover, a hidden hematopoietic cell can be caught to unlock a minigame to earn gold coins. A wrong answer to a skill question makes SUPER HEMO lose the patient’s trust and lose gold coins, and a wrong answer to a treatment question makes the hero lose the patient’s trust. Lady Stem Cell gives immediate feedback to every question, thus maintaining the user’s motivation and commitment to the game [24]. If all gold coins and the patient’s trust are lost, Mister Insurance takes SUPER HEMO back to the beginning of the case, where the player can immediately play again if they have enough gold coins; otherwise, they can still earn gold coins in minigames before trying the case again. The hidden collected cells constitute an atlas of hematopoietic cells that contains a precise description and a real picture (taken by microscopy) of each blood cell. Minigames are always related to hematology in a fun way.

When SUPER HEMO reaches 80% success in the world’s 5 clinical cases (and achieves a total of 12 stars of a possible 15) the player wins a new asset (a myelogram) and is thereby allowed to move on to a higher level, which corresponds to another hematopoietic world.

Throughout the game, to maintain the dreamlike atmosphere and immersion, different theme songs accompany the player (with the possibility to mute them). The player also has the option to reset the game on the home screen (not pictured).

### Development

#### Technology

The game was programmed in January 2019 using C# in the Unity game engine, for computers only (ie, there was no mobile app). The invention was protected with an Inter Deposit Digital Number on September 9, 2020, for the University of Lorraine. To date, the game is available in French only and is free of charge at the University.

#### Video Game Beta Tests

Three beta-test sessions were organized with small groups of undergraduate and graduate physician and pharmacy volunteer students. The goal was to obtain a primary evaluation of the game design and concept and to debug the game. The volunteers played the game for 1 hour and were then asked to fill in a questionnaire (Multimedia Appendix 3) about several aspects of the game, including the graphical interface, gameplay, use of multimedia, and educational content, and provide ideas for improvement and general comments. We collected the questionnaires and analyzed the answers to modify or improve the game and the teaching methods, if required.

### Formative Evaluation

#### Ethics Approval

The study was conducted in accordance with the Helsinki Declaration and Resolution. The study was approved by the Pedagogic Committee of the Faculté de Pharmacie, Université de Lorraine (June 17, 2021) and Faculté de Médecine, Université de Lorraine (July 7, 2021).

#### Recruitment and Game Evaluation

Students in their third year of medicine and pharmacy courses were identified as the most appropriate study participants, since hematology is a regular and mandatory course unit during this academic year. These student cohorts were expected to benefit the most from additional exposure to clinical learning while using SUPER HEMO, as their upcoming final exams were planned to take place shortly after game exposure. The game evaluation was performed after 2 weeks of classical hematology teaching. The gaming course consisted of 1.5 hours of gaming (in the Red World) that covered the following standard red blood cell disorders: anemia and polycythemia. The game evaluation was split into 2 phases examining (1) the immediate knowledge acquisition of the players and (2) whether the players successfully completed their final hematology examination.

First, to evaluate the effectiveness of this teaching method, voluntary participants from each course were asked to complete a knowledge test consisting of 10 multiple-choice questions (Multimedia Appendix 4); one point was obtained for each correct answer. The participants completed the 10-question test before (pretest) and after playing the game for 1 hour (posttest). An online questionnaire was designed to assess playability and the students’ understanding of SUPER HEMO. Students were also asked to rate their level of confidence after the gaming session on a questionnaire. Qualitative data considering the students’ general feedback was also collected. All answers were anonymized.

Second, successful completion of the final hematology examination was extracted for students who had participated in SUPER HEMO and those who had not by the pedagogic committee, and mean results were compared.

#### Statistics

Statistical analyses were carried out using Prism (version 5.0; GraphPad). Comparisons of participation rates for the physician and pharmacy students, as well as the female to male ratio of the groups, were made with the Fisher exact test. Pre- and posttest scores and the final evaluation were expressed as median values, with the range and 25th to 75th percentiles. Pre- and posttest results were compared using a paired Wilcoxon signed-rank test in the 3 groups (ie, the overall population, the physician students, and the pharmacy students). Final evaluation results were compared using the Mann-Whitney test. As participation was on a voluntary basis, a post hoc power analysis of the final evaluation’s scores was also performed.

## Results

### Beta Tests

**Figure 3:**
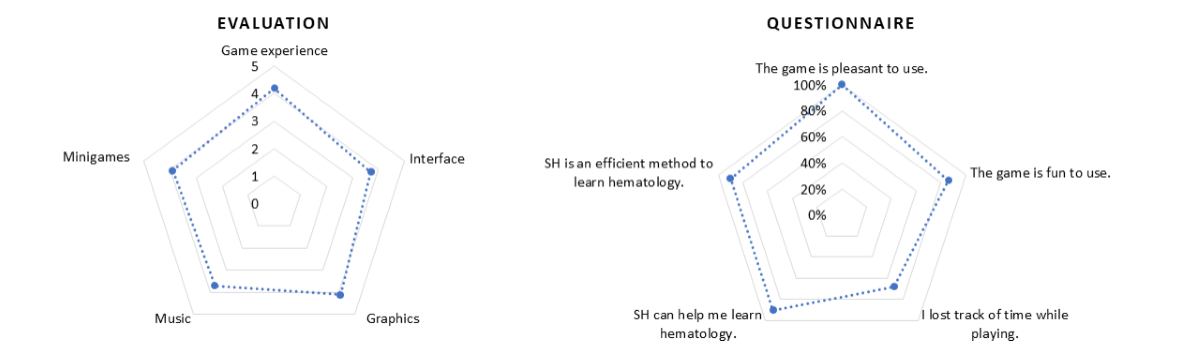
Beta-test results. SH: SUPER HEMO.

The results of the 3 beta tests (Figure 3) indicated a significant interest in this new SG.

The interface received a score of 3.7 of 5. As for the multimedia aspect, the graphics and music received scores of 4.1 and 3.7 of 5, respectively. All students stated that they enjoyed the game, 86% (19/22) found the game fun to use, and 68% (15/22) even lost track of time while playing. Regarding the educational content, 90% of the students (20/22) found that SUPER HEMO represented an efficient method to learn hematology.

The beta tests allowed us to detect a few points to improve SUPER HEMO and address issues encountered by the students. Some students had difficulties answering certain types of questions (for example, ones that used drag-and-drop), or they could not find the minigames. This led us to integrate tutorials at the beginning of each game, such as on how to answer questions encountered for the first time. We also modified some questions and minigame rules to better drive the players. Moreover, some players forgot the CBC results while talking to the unwell characters and frequently wanted to check them; therefore, we added a button to allow the players to check the CBC results as often as they wanted to. Finally, as the game tasks could be interrupted, we added a “log info” button to remind the students of dialogue text.

The free comments included mostly thanks for the initiative and anticipation for the sequels.

### Integrated Teaching Approach in Addition to the Standard Hematology Undergraduate Curriculum

A total of 153 volunteer students were recruited, including 86 of 324 physician students (26.5%) and 67 of 115 pharmacy students (58%), who agreed to participate in this complementary session at the end of the standard lecture-based hematology course. Of note, the proportion of female students was higher in the participant group (Table 1).

**Figure 4:**
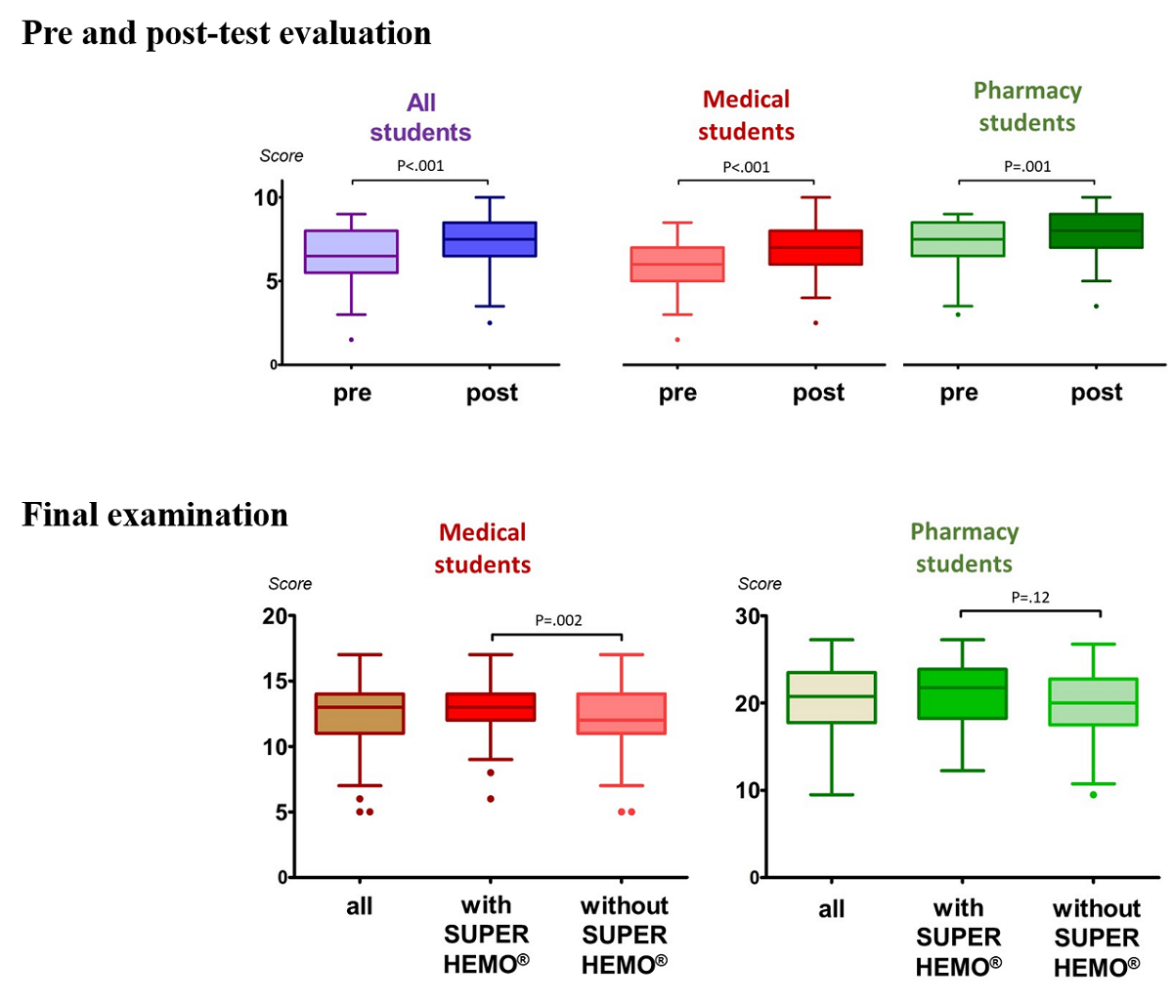
SUPER HEMO evaluation.

Overall, the volunteer students were evaluated on a 10-point scale (Table 2 and Figure 4). They had a higher posttest score (median 7.5, range 2.5-10) than pretest score (median 6.5, range 1.5-9; *P*=.001), indicating that the game slightly improved their immediate knowledge acquisition. The median pre-and posttest scores were 6 (range 1.5-8) and 7 (range 2.5-10), respectively, for the physician students (*P*<.001) and 7.5 (range 3-9) and 8 (range 3.5-10), respectively, for the pharmacy students (*P*<.001).

At the final examination (Figure 4), we observed that the physician students who played SUPER HEMO obtained a slightly higher score (median 13/20, range 6-17) than those who did not play SUPER HEMO (median 12/20, range 5-17; *P*=.002), with a satisfactory study power (83%). The pharmacy students who played SUPER HEMO had a score (median 21.75/30, range 12.25-27.25) that was not statistically significantly different from those who did not play SUPER HEMO (median 19.8/30, range 9.5-26.75; *P*=.12). Unfortunately, the study power was low (64%) for this group.

**Table 1:** Comparison of participants and nonparticipants in SUPER HEMO evaluation.

Characteristics		Participants, n	Nonparticipants, n	*P* value	
**All students (N=439)**					<.001
	Female	110	161		
	Male	43	125		
**Physician students (n=324)**					.05
	Female	60	136		
	Male	26	102		
**Pharmacy students (n=115)**					.02
	Female	50	25		
	Male	17	23		

**Table 2:** Pre- and posttest evaluations (scores are on a 10-point scale).

	Pretest				Posttest				*P* value
	Median score	Range	25th-75th percentile	Median score		Range	25th-75th percentile		
All students	6.5	1.5-9	5.5-8	7.5		2.5-10	6.5-8.5	<.001	
Physician students	6	1.5-8	5-7	7		2.5-10	6-8	<.001	
Pharmacy students	7.5	3-9	6.5-8.5	8		3.5-10	7-9	.001	

### Gameplay Satisfaction

**Figure 5:**
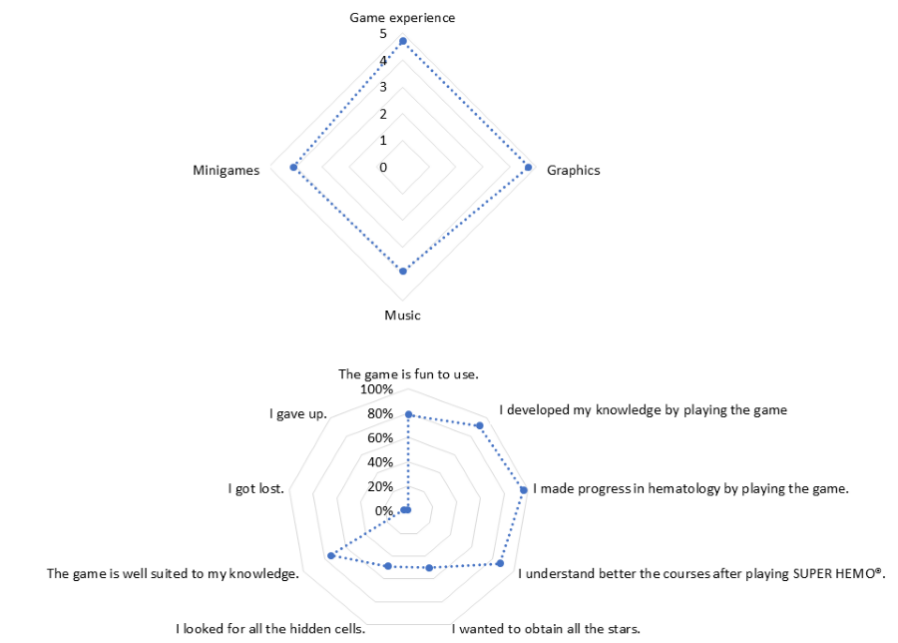
Gameplay satisfaction.

Among 153 volunteer students, 143 answered the questionnaire (93.4%). Game experience received a score of 4.7 out of 5, and 79% of the students (n=113) found the game fun to use. Regarding multimedia, graphics received a score of 4.7 out of 5, and music received 3.9 out of 5. The minigames were scored as attractive (4.1/5; Figure 5).

Concerning the educational content, most of the 153 students indicated that they had augmented their knowledge (n=130, 91%), had made progress in hematology (n=137, 96%), and better understood their courses (n=124, 87%) after playing SUPER HEMO. In addition, 71 students (50%) indicated that they specifically aimed to obtain the top 3 stars at the end of each case in the game, and 70 students (49%) dedicated specific effort to collect all the hidden cells. While the effect of rewards on memory appears well documented, it has recently been reported that incentives can also have counterproductive effects on memory [25]. Our videogame reward system was, however, developed to create a realistic game environment with well-known reinforcement and reward schedules. Of the 153 participants, 106 (74%) indicated that the flow of the game suited their knowledge, zero indicated they were bored by the game, 5 (3%) became lost in the game, and only 1 gave up.

## Discussion

### Principal Findings

SUPER HEMO was developed to increase students’ motivation for learning hematology. In fact, as previously described, when students feel involved, they are more likely to achieve educational goals [26]. But with only 4 hematology teachers at the university, bedside (for clinical symptoms) and laboratory (for cell recognition) teaching in undergraduate medical education could not be fully accomplished for that year’s 324 physician and 115 pharmacy students. Consequently, SUPER HEMO was developed to confront students with different clinical situations, improve their cell observation skills, and supplement the classical teaching model. Because players may have different skills, we developed the pedagogic content concomitantly with gamification [27]. Special characters, such as Lady Stem Cell, were created to regularly debrief the players on wrong or right answers to questions [28]. SUPER HEMO level 1 (the Red World) was developed and beta tested over a 2-year period. Subsequently, SUPER HEMO’s approach was integrated to the standard hematology undergraduate curriculum of third-year physician and pharmacy students and the SG was evaluated.

In health care education, studies comparing SGs to other teaching methods with prospective evaluations are scarce [29,30]. We assumed that this prospective evaluation might be useful to justify SUPER HEMO’s integration with the standard hematology curriculum. The satisfaction questionnaire clearly showed that most students enjoyed playing SUPER HEMO (113/142, 80%) and felt that they learned from it (123/142, 86%). These results demonstrate the students’ strong commitment to the game. This result was reinforced by the free comments (eg, “I find the interface truly attractive; it really makes me want to do well” and “A good experience, dialogs or context are quite funny but still remain consistent!”). Several students inquired about playing the rest of the game (eg, “We’re looking forward to the white and yellow worlds’ release” and “I hope I can play again soon!”). Students highlighted the link between the game and seriousness (eg, “It allowed me to study in a fun way” and “Fun way to learn hematology or to practice without the feeling that I have studied”).

Knowledge improvement with this complementary method to the standard course was more difficult to evaluate. If a positive effect of SUPER HEMO was clearly observed for short-term knowledge (ie, the results of the pre- and posttests), the measurement of learning outcome (ie, the results of the final examination) should also be discussed. First, the first SUPER HEMO session ran through the COVID-19 pandemic, when restrictions were in place on attending courses. Therefore, we could not evaluate the effectiveness of this SG with a randomized controlled trial. After discussion with the pedagogic committee during the pandemic, courses were transmitted virtually to students who wanted to stay at home; less than 10% of students attended on site. In order not to penalize medically or psychologically fragile students, we decided to invite students to participate as they chose to do so. We can hypothesize that volunteer students who came to the SUPER HEMO session were the most motivated in the class, thus biasing the results of the final exams. Second, we observed that pharmacy students were more likely to attend the SUPER HEMO session than the physician students. Of note, the pharmacy students were probably more motivated, as their final exam took place a few days after the gaming session, whereas the physician students had their final exam 1 month after the gaming session. For the pharmacy students, although the participation rate was satisfactory, the study was underpowered, suggesting that follow-up studies (within the next few years) with more students might confirm that playing SUPER HEMO allowed students to obtain a better score in the final hematology evaluation (as was observed for the physician students). Third, our conclusions on interest in SUPER HEMO should be moderated by consideration of the SUPER HEMO session design. The session had a time limit of 90 minutes and was based only on the available “Red World,” which dealt with anemia and polycythemia, while the final evaluation contained questions on red blood cells, white blood cells, and platelets. We therefore propose that in the future (1) three worlds corresponding to the three lineages of hematopoietic cells in the CBC be made available to encompass the whole hematology program, (2) game-based learning should have no time restrictions, and (3) new evaluations with more participants should be organized with other universities.

Currently, we consider that our research contributes to the literature by providing a new game for teaching hematology and an investigation of the effectiveness of the SG context for hematology learning. This first experiment with SUPER HEMO commits us to develop this SG for hematology in future academic years. Future work will focus on developing levels 2, 3, and 4, the “White World,” “Yellow World,” and “Complex World,” respectively, with all parameters varying in the latter. We propose an evolving game that covers the entire program of hematology and is accessible on the digital platform of the University of Lorraine. SGs have been poorly developed for hematology education. Tan et al [21] were the first to report an SG, developed for nurses in Singapore, that dealt with the safe administration of blood transfusions. This SG was prospectively tested with 103 second-year undergraduate nursing students randomized into control or experimental groups. Posttest knowledge and mean scores for confidence improved significantly in the experimental group (*P*<.001) after the SG intervention compared to mean pretest scores and mean posttest scores for the control group (*P*<.001). However, no significant differences (*P*=.11) were found between the experimental and control groups for mean posttest performance scores. Four years later, the participants evaluated the SG positively as an innovative and stimulating learning tool; however, more rigorous efforts to improve the interface and alleviate technical issues were suggested as ways to make the SG more accessible and intuitive for all levels of nursing staff [22]. To our knowledge, SUPER HEMO is the first SG developed for CBC interpretation to be used by teachers of hematology for students in medicine, pharmacy, dentistry, and midwifery. During the COVID-19 pandemic, education was suddenly disrupted, and universities were mandated to switch to online teaching. The game-based computer app that we designed represents a potentially effective teaching tool for health care students.

### Conclusion

This study has provided evidence that SUPER HEMO met our primary objective: to develop an SG for hematology that was playable and acceptable overall. The usability of SUPER HEMO was demonstrated beyond the initial beta-testing pilot study; we obtained preliminary evidence that SUPER HEMO might be a useful educational tool. We will use it as a supplement to lecture-based courses and trace the time and frequency of logins to SUPER HEMO. Last, we propose to correlate this continuous training to student results for the third-year final exams, final graduation exams (at the end of the fifth year), and for the student’s specialty choice.

## Appendix

Multimedia Appendix 1 Red Cell World.

Multimedia Appendix 2 SUPER HEMO avatars speaking with different characters.

Multimedia Appendix 3 Beta-test questionnaire.

Multimedia Appendix 4 Pre and post tests.

## Abbreviations

CBC: complete blood count
SG: serious game
