# Supplementary figures and images for: A Serious Game About Hematology for Health Care Workers (SUPER HEMO): Development and Validation Study
Source: JMIR Serious Games. 2023 Feb 13;11:e40350. doi: 10.2196/40350 (PMC9972200; doi:10.2196/40350)

## Slide 1
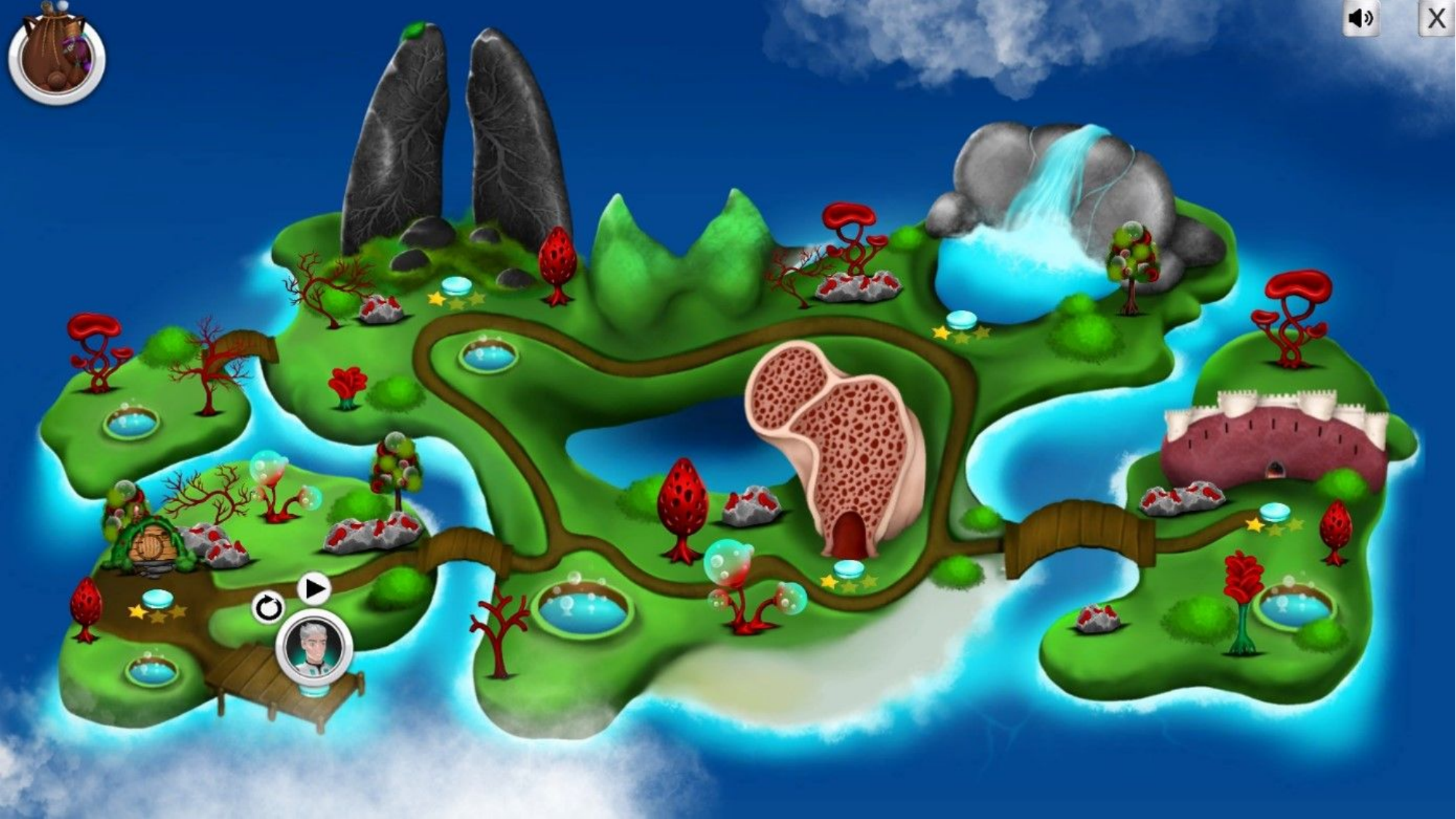

Supplement: Multimedia Appendix 1 [file games_v11i1e40350_app1.pptx]

## Slide 1
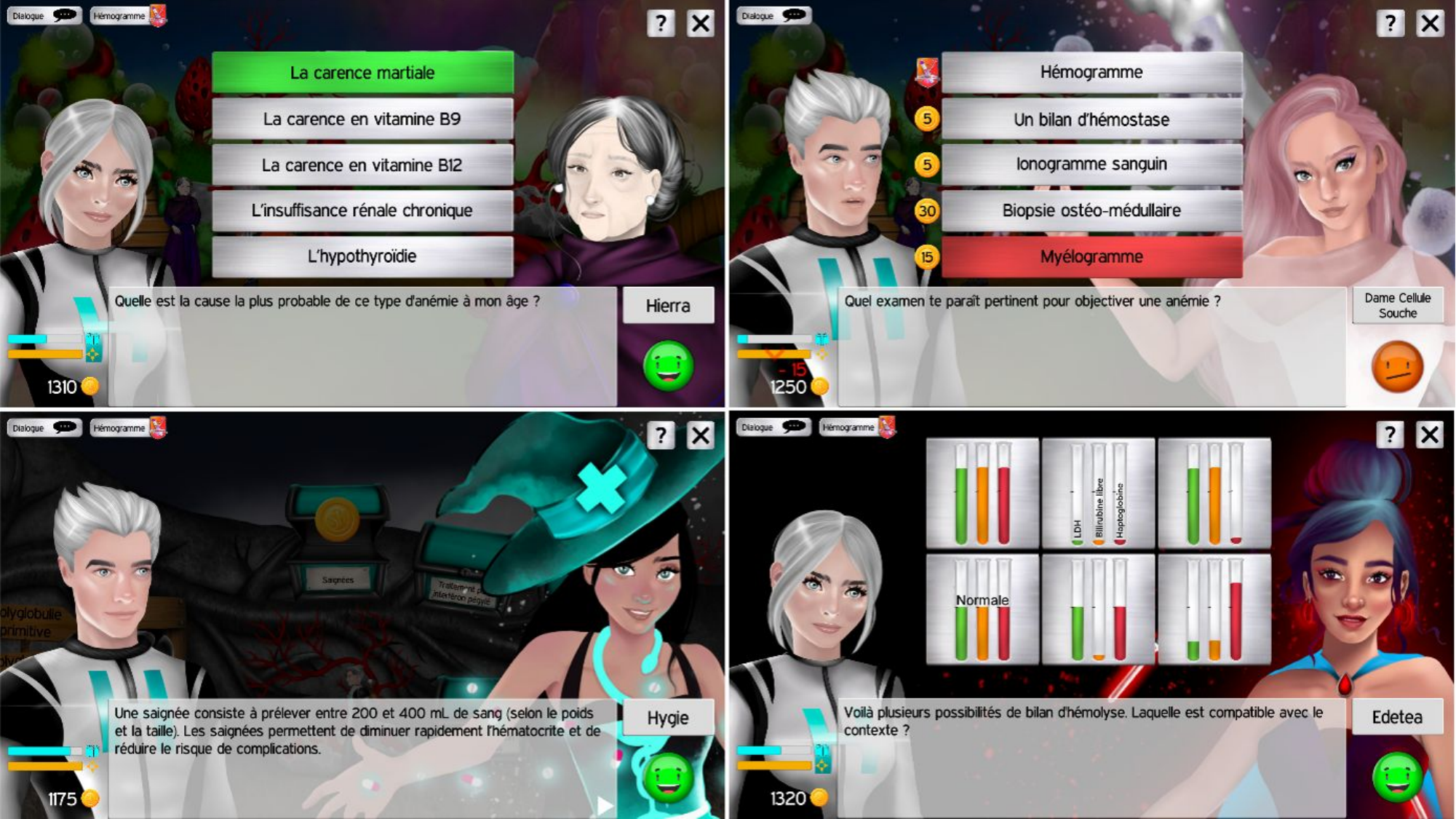

Supplement: Multimedia Appendix 2 [file games_v11i1e40350_app2.pptx]
