# Supplementary material for: A Serious Game About Hematology for Health Care Workers (SUPER HEMO): Development and Validation Study
Source: JMIR Serious Games. 2023 Feb 13;11:e40350. doi: 10.2196/40350 (PMC9972200; doi:10.2196/40350)
Supplement: Multimedia Appendix 3 [file games_v11i1e40350_app3.pdf]

Beta-test questionnaire

Q1. On a 5-point scale, how would you rate your game experience?

1 (bad) 2 3 4 5 (excellent)

Q2. On a 5-point scale, how would you rate the graphisms?

1 (ugly) 2 3 4 5 (beautiful)

Q3. On a 5-point scale, how would you rate the music?

1 (annoying) 2 3 4 5 (well suited)

Q4. On a 5-point scale, how would you rate the mini-games?

1 (bad) 2 3 4 5 (excellent)

Q5. On a 5-point scale, how would you rate the interface?

1 (unclear) 2 3 4 5 (clear)

Q6. Do you agree to the sentences below?

	Strongly disagree	Disagree	Neutral	Agree	Strongly agree
The game is fun to use.					
The game is pleasant to use.					
I lost track of time while playing.					
SUPER HEMO® is an efficient method to learn hematology.					
SUPER HEMO® can help me learn hematology.					
