# Supplementary material for: A Serious Game About Hematology for Health Care Workers (SUPER HEMO): Development and Validation Study
Source: JMIR Serious Games. 2023 Feb 13;11:e40350. doi: 10.2196/40350 (PMC9972200; doi:10.2196/40350)
Supplement: Multimedia Appendix 4 [file games_v11i1e40350_app4.pdf]

THE PRE AND POST TEST

1. Common symptoms such as : pallor, dyspnea, fatigue and dizziness +/- headaches are related to

A.Polycythemia

B.Anemia

C.Hyperleukocytosis

D.Thrombocytosis

E.Neutropenia

2. Which routine screening test do you need for anemia diagnosis?

A. Complete blood count (CBC)

B. Bone marrow smear

C. Bone marrow biopsy

D. Coagulation screen

E. Reticulocyte count

3. Here are the results of a complete blood count (CBC):

Red blood cells 3.9 10^{12}/L

Hemoglobin: 61 g/L

Hematocrit: 24.2%

Mean corpuscular volume (MCV): 62 fL

Mean corpuscular hemoglobin (MCH): 15 pg

Mean corpuscular hemoglobin concentration (MCHC): 25 g/dL

Which parameter does define anemia?

A.Red blood cell count

B.Hemoglobin

C.Hematocrit

D.Mean corpuscular volume

E.Mean corpuscular hemoglobin concentration

4. According to your knowledge, anemia in this CBC, can be characterized as

A. Microcytic and hypochromic

B. Macrocytic and hypochromic

C. Normocytic and hyperchromic

D. Normocytic and normochromic

E. Microcytic and normochromic

5.For this anemia, what do you analyze first to determine its underlying etiology?

A. Reticulocyte count

B. Mean corpuscular volume

C. Bone marrow smear

D. Hemolysis profile

E. Serum creatinine

6. What medical conditions cause microcytic anemia?

A. Iron-deficiency

B. Chronic inflammation

C. Thyroid dysfunction

D. Chronic kidney failure

E. Folate deficiency

Q7. Here are partial results of a complete blood count (CBC):

Red blood cells 2 10^{12}/L

Hemoglobin: 70 g/L

Hematocrit: 25%

After calculation, you can tell that this anemia is

A.Macrocytic and normochromic

B.Normocytic and normochromic

C.Normocytic and hyperchromic

D.Normocytic and hypochromic

E.Microcytic and normochromic

Q8 .For normochromic and normocytic anemia, which diagnostic tests do you prescribe first ?

A.Reticulocyte count

B.Iron profile

C.Folate profile

D.Hemolysis profile

E.Serum creatinine

Q9. In hemolytic anemia, what is correct ?

A.reticulocyte count is lower than 60.10^9/L

B.reticulocyte count is upper than 120.10^9/L

C.Haptoglobin is low

D.Mean corpuscular volume is often lower than 80fL

E. Low ferritin

Q10. In normocytic normochromic anemia with decreased bone marrow red cell production , which diagnosis could you propose?

A.Sickle cell anemia

B.Chronic respiratory failure

C.B12 vitamin deficiency

D.Chronic kidney failure

E. Iron deficiency
